# Adequate Levels of Adherence with Controller Medication Is Associated with Increased Use of Rescue Medication in Asthmatic Children

**DOI:** 10.1371/journal.pone.0039130

**Published:** 2012-06-27

**Authors:** Hajer Elkout, Peter J. Helms, Colin R. Simpson, James S. McLay

**Affiliations:** 1 Department of Child Health, Division of Applied Health Sciences School of Medicine, University of Aberdeen, Aberdeen, United Kingdom; 2 Department of General Practice and Primary Care, Division of Applied Health Sciences School of Medicine, University of Aberdeen, Aberdeen, United Kingdom; 3 Centre for Population Health Sciences, Foresterhill Health Centre, University of Edinburgh, Edinburgh, United Kingdom; California Pacific Medicial Center Research Institute, United States of America

## Abstract

**Background:**

The role of asthma controller medication adherence and the level of asthma control in children is poorly defined.

**Aims:**

To assess the association between asthma controller medication adherence and asthma control in children using routinely acquired prescribing data.

**Methods:**

A retrospective observational study of children aged 0–18 years prescribed inhaled corticosteroids only (ICS), leukotriene receptors antagonists (LTRA), or long-acting β2 agonists (LABA) and ICS prescribed as separate or combined inhalers, between 01/09/2001 and 31/08/2006, registered with primary care practices contributing to the Practice Team Information database. The medication possession ratio (MPR) was calculated and associations with asthma control explored. Poor asthma control was defined as the issue of prescriptions for ≥1 course of oral corticosteroids (OCS) and/or ≥6 short-acting β2 agonists (SABA) canisters annually.

**Results:**

A total of 3172 children prescribed asthma controller medication were identified. Of these, 15–39% (depending on controller medication) demonstrated adequate MPR. Adequate MPR was associated with male gender, good socio-economic status, and oral LTRA therapy. Adequate MPR was more likely to be associated with increased use of rescue medication. However logistic regression only identified a significant relationship for ICS only (odds ratio [OR], 1.89; 95% confidence interval [CI], 1.35–2.48; p<0.001), LTRA (OR, 2.11; 95% CI, 1.27–3.48; p = 0.004) and LABA/ICS (OR, 2.85; 95% CI, 1.62–5.02; p<0.001).

**Conclusion:**

Poor adherence was observed for all asthma controller medications, although was significantly better for oral LRTA. In this study adequate adherence was not associated with the use of less rescue medication, suggesting that adherence is a complex issue.

## Introduction

Asthma medications are among the most commonly prescribed medicines for children in the community [1], however despite the availability and proven efficacy of such medications, asthma remains a major cause of morbidity. Scottish health survey data for 2010 confirms that despite an overall decrease in the annual incidence in children of both wheeze and asthma, they remain common childhood complaints with 22% and 13% of children aged 0–15 having a history of wheeze, and a medical diagnosis of asthma respectively [2]. Asthma management in children can be challenging for a number of reasons, including limited outcome data, inappropriate prescribing and poor adherence with prescribed therapy [3]–[7].

It is generally recognized that adherence with prescribed medications in children is poor, with less than 50% using their asthma controller medication as prescribed [8]–[9], which in turn is associated with poor asthma control [5]–[7]. There are, however, few studies which have investigated the relationship between asthma control and adherence in children [5]–[7], [10]–[12].

The aims of this study were to assess the association between the levels of adherence with asthma controller medication and asthma control using routinely collected primary care prescribing data.

## Methods

### Study Population

This observational retrospective study used data from the Practice Team Information (PTI) database [13]. Children who were issued a new prescription for one of the four following classes of asthma controller medications were identified; inhaled corticosteroids only (ICS), leukotriene receptors antagonists (LTRA), long-acting β_2_ agonists (LABA) and ICS prescribed concurrently as separate inhalers (LABA+ICS) or as a fixed-dose combination (LABA/ICS), between 01/09/2001 and 31/08/2006. The date of the first prescription was regarded as the index date. To be included in the study, a child was required to be registered in the database for at least a year before and a year after the index prescription date.

### Measures of Adherence

Adherence with asthma controller medication was assessed using the medication possession ratio (MPR), a methodology which is commonly used in adherence research [7]–[8], [14]–[18].

The MPR is a measure of medication availability and is calculated as the total number of days’ supply of medication prescribed divided by the total number of days in the follow up period, multiplied by 100, and expressed as a percentage:
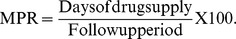



where days of drug supply equals the number of days a prescription should last based on the dosing instructions of the prescriber (Days of drug supply  =  number of doses in a prescription divided by the dosing frequency). The follow up period is the interval between first and last prescription for that patient. Since at least two prescriptions were required to calculate the follow-up period, only children with two or more consecutive prescriptions were included in the study. For children prescribed LABA+ICS, days of ICS drug supply were calculated.

Ideally, a 200 dose inhaler, prescribed as one puff twice daily, should last 100 days and a repeat prescription requested after approximately 100 days, i.e. MPR = 100%. Requesting a repeat prescription after or before 100 days would result in undersupply or oversupply. In the present study, adequate MPR was defined as drug supply covering 80–120% of the duration of prescribed treatment, a range that has been used previously to evaluate both low adherence and stockpiling of therapy [15].

### Outcome Measures

The level of asthma control was assessed by identifying both the number of short-acting β_2_ agonist (SABA) inhalers and/or courses of oral corticosteroids (OCS) prescribed annually during the study period. Prescription of more than 6 canisters of SABA and/or at least one OCS rescue course annually was used as an indication of poor control [5]–[6], [13], [16].

### Statistical Analyses

Descriptive statistics were used to determine baseline population characteristics. MPR was dichotomised as either adequate (MPR between 80–120% inclusive) or inadequate (MPR outwith the 80–120% range). MPR association with age (grouped into 0–4, 5–11 and >11 years age bands), gender, socio-economic status (Scottish Index of Multiple Deprivation (SCSIMD) dichotomized into low, 0–5 and high, 6–10) and post index prescribing of SABA (<6 canisters vs. ≥6) and OCS (none vs. ≥1) was assessed. To confirm relationships between adequate MPR and asthma control, associations were explored using the chi-square test followed by multivariate logistic regression analysis adjusting for age, gender, socio-economic status and prescribing of other asthma medications. All analyses were performed using SPSS (SPSS for Windows V.17.0). Where appropriate a two sided t test was used and a p value of <0.05 was considered significant. Adjusted odds ratios (OR) and 95% confidence intervals (CI) were reported for multivariate logistic regression results.

## Results

We identified 53,736 prescriptions for the controller medications of interest issued during the study period. The MPR could be calculated for 92% of these prescriptions. The final cohort included 3172 children of whom 2297 were prescribed ICS alone, 394 LTRA, 481 fixed dose combination LABA/ICS, and 219 concurrent LABA+ICS (Table 1).

**Table 1:** Baseline characteristics of children prescribed different asthma controller medications.

Characteristic	ICS onlyN = 2297N %	LTRAN = 394N %	LABA/ICSN = 481N %	LABA+ICSN = 219N %
**Mean age, years**	6	6	9	8
**Male (%)**	1357 (59.0)	227 (57.6)	256 (53.2)	126 (57.5)
**scsimd10 (%)**				
Low	1048 (55.7)	166 (42.1)	251 (52.1)	75 (34.2)
High	1249 (44.3)	228 (57.9)	230 (47.9)	144 (65.8)
**Pre-index SABA (%)**				
<6 canisters/year	1311 (95.7)	269 (74.9)	342 (77.5)	136 (75.9)
6–9 canisters/year	41 (3.1)	58 (16.2)	63 (14.4)	26 (14.7)
9 canisters/year	17 (1.2)	32 (8.9)	36 (8.1)	17 (9.4)
Pre-index OCS (%)	212 (9.2)	116 (29.4)	111 (23.0)	71 (32.4)

*ICS =  Inhaled corticosteroid; LTRA = leukotriene receptors antagonists; LABA/ICS =  fixed dose long-acting β2 -agonist and inhaled corticosteroids combination; LABA+ICS =  concurrent long-acting β2 -agonist and inhaled corticosteroids separate inhaler; SCSIMD  =  Scottish Index of Multiple Deprivation; SABA =  Short-acting β2–agonist; OCS =  Oral corticosteroids.*

### Adherence Rates

**Figure 1:**
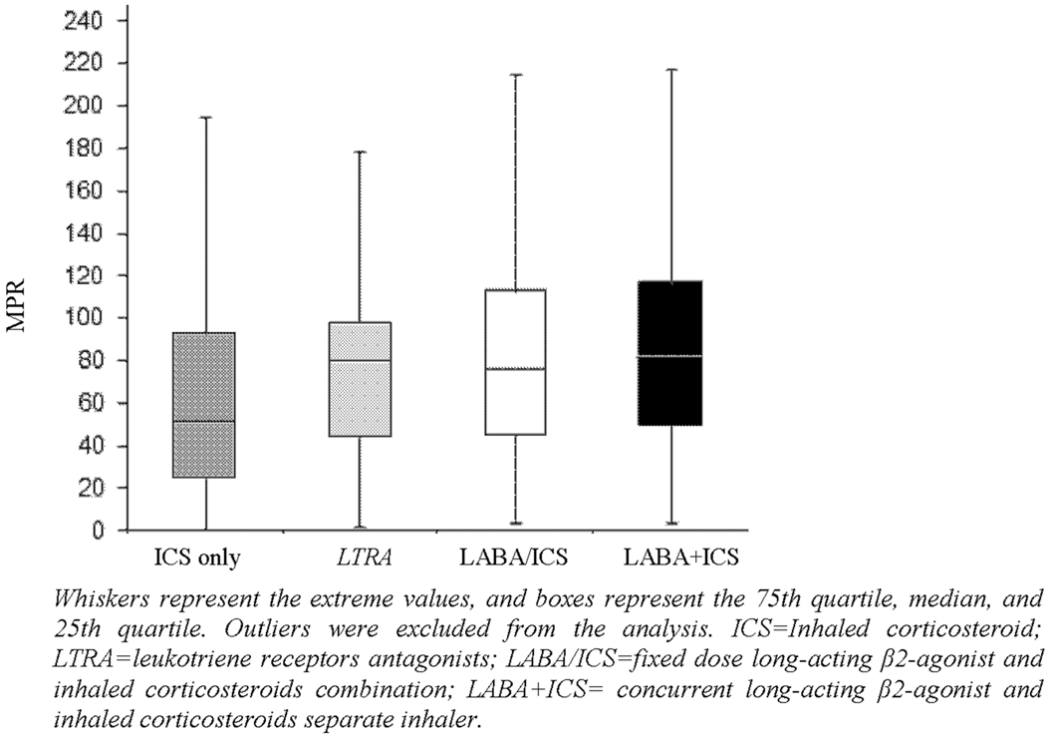
Box plot for the distribution of Medication Possession Ratio of prescribed asthma controller medications. Whiskers represent the extreme values, and boxes represent the 75th quartile, median, and 25th quartile. Outliers were excluded from the analysis. MPR =  Medication possession ratio; ICS = Inhaled corticosteroid; LTRA = leukotriene receptors antagonists; LABA/ICS = fixed dose long-acting β2-agonist and inhaled corticosteroids combination; LABA+ICS =  concurrent long-acting β2-agonist and inhaled corticosteroids separate inhaler.

For the medications of interest, the MPR was poor, ranging from close to 0 to over 200% (Figure 1). Depending on the controller medication, between 15–39% of children had an adequate MPR (defined as MPR between 80–120%). The proportion of children with an adequate MPR was significantly greater for LTRA when compared with other controller medications (39%, p<0.05). Oversupply (MPR>120%) was observed in 9–21% of the study group and was greatest for those prescribed concurrent LABA+ICS (Table 2, Figure 1). Under supply (MPR<80%) was more common than oversupply and was observed in 51–69% of the study population. Undersupply was significantly greater in children prescribed ICS only when compared with other controller medication (p<0.001, Table 2, Figure 1).

**Table 2:** Medication Possession Ratio for different asthma controller medications.

Variable	ICS only	LTRA	LABA/ICS	LABA+ICS
Adequate MPR (%)	356 (15)	156 (39)	123 (25)	62 (28)
Under supply (%)	1574 (69)	204 (52)	260 (54)	113 (51)
Over supply (%)	367 (16)	34 (9)	98 (21)	44 (21)
Mean (SD)	70 (71)	75 (40)	93 (78)	91 (89)
Median (IQR)	51 (25–92)	70 (44–98)	88 (45–112)	82 (49–115)
Total	2297	394	481	219

*ICS =  Inhaled corticosteroid; LTRA = leukotriene receptors antagonists; LABA/ICS =  fixed dose long-acting β2 -agonist and inhaled corticosteroids combination; LABA+ICS =  concurrent long-acting β2 -agonist and inhaled corticosteroids separate inhaler; MPR = Medication possession ratio; SD =  Standard deviation; IQR; Inter-quartile range.*

### Factors Influencing Adherence Rates

Adequate MPR (80–120%) was higher in boys than girls, better in children with higher SCSIMD scores, and significantly higher for children prescribed oral LTRA when compared to other controller medications (Tables 1,2). Adequate MPR was also significantly greater in younger children aged <5 managed on ICS as their only controller medication (p<0.001) when compared to older children and adolescents prescribed the same regimen (Table 3).

**Table 3:** Adequate MPR for asthma controller medications by deprivation index, age and gender.

Controllertreatment	MPR 80–120%						
	SCSIMD		Gender		Age bands		
	0–5	6–10	Girls	Boys	0–4 years	5–11 years	>11 years
ICS only	153 (44.0)	195 (56.0)	142 (41)	206 (59)	170 (49)*	135 (39)*	43 (12)*
LTRA	69 (43.7)	89 (56.3)	69 (44)	87 (56)	72 (46)	69 (44)	15 (10)
LABA/ICS	67 (53.2)	59 (46.8)	54 (44)	69 (56)	17 (14)	77 (62)	29 (24)
LABA+ICS	30 (48.8)	32 (51.6)	23 (37)	39 (63)*	8 (13)	43 (69)	11 (18)

* *P<0.001; ICS =  Inhaled corticosteroid; LTRA = leukotriene receptors antagonists; LABA/ICS =  fixed dose long-acting β2 -agonist and inhaled corticosteroids combination; LABA+ICS =  concurrent long-acting β2 -agonist and inhaled corticosteroids separate inhaler; SCSIMD  =  Scottish Index of Multiple Deprivation.*

### Medication Possession Ratio vs Asthma Control

Significantly more children with adequate MPR were prescribed >6 SABA canisters per year when compared to those with an inadequate MPR for all study medications. However, multivariate logistic regression confirmed this association for ICS only, LTRA and LABA/ICS (Tables 3,4). More children with adequate MPR were also prescribed OCS in the post index year, when compared to those with inadequate MPR, this difference failed to reach statistical significance (Table 5). Multivariate logistic regression analyses demonstrated a similar relationship between adequate MPR and OCS.

**Table 4:** The relationship between reliever medication prescription in the post index year and the medication possession ratio.

Reliever medication	ICS only		LTRA		LABA/ICS		LABA+ICS	
	Adequate MPR	Inadequate MPR	Adequate MPR	Inadequate MPR	Adequate MPR	Inadequate MPR	Adequate MPR	Inadequate MPR
**SABA**								
<6 canister/year	230 (76.9)*	1529 (85.4)	59 (62.8)**	235 (77.3)	44 (62.0)*	338 (82.8)	37 (57.8)**	123 (65.1)
6–9 canister/year	55(18.4)*	190 (10.6)	23 (24.5)**	44 (14.5)	16 (22.5)*	47 (11.5)	16 (25.0)**	45 (23.8)
>9 canister/year	14 (4.7)*	72 (4.0)	12 (12.8)**	25 (8.2)	11 (15.5)*	23 (5.6)	11 (17.2)**	21 (11.1)
**OCS** (≥1 course/year )	54 (18.1)	262 (14.6)	27 (28.7)	70 (23.0)	14 (19.7)	71 (17.4)	21 (32.8)	48 (25.4)

*% of children with adequate vs inadequate MPR. e.g. x% of children with adequate MPR had an OCS prescription while xx% of those with inadequate MPR had an OCS prescription.*

* *P<0.001;*

** *P<0.05.*

*SABA =  Short-acting β2–agonist; OCS =  Oral corticosteroids; ICS =  Inhaled corticosteroid; LTRA = leukotriene receptors antagonists; LABA/ICS =  fixed dose long-acting β2 -agonist and inhaled corticosteroids combination; LABA+ICS =  concurrent long-acting β2 -agonist and inhaled corticosteroids separate inhaler.*

**Table 5:** Adjusted* odds ratios of being prescribed reliever medications for children with adequate versus inadequate MPR, stratified by controller medication.

Outcomes	Adjusted OR*	*P*-value	95% CI
At least one OCS course			
ICS only	1.02	0.18	1.00–1.04
LTRA	1.34	0.26	0.79–2.27
LABA/ICS	1.12	0.53	0.58–2.11
LABA+ICS	1.43	0.27	0.75–2.71
≥6 SABA canisters			
ICS only	1.89	<0.001	1.35–2.48
LTRA	2.11	0.004	1.27–3.49
LABA/ICS	2.85	<0.001	1.62–5.02
LABA+ICS	1.45	0.22	0.79–2.65

* *Adjusted for age, gender, socio-economic status and pre index prescribing of asthma medications. Adjusted OR = Adjusted odds ratios; SABA = Short-acting β2–agonist; OCS = Oral corticosteroids; ICS = Inhaled corticosteroid; LTRA = leukotriene receptors antagonists; LABA/ICS = fixed dose long-acting β2-agonist and inhaled corticosteroids combination; LABA+ICS = concurrent long-acting β2 -agonist and inhaled corticosteroids separate inhaler.*

Because the proportion of children oversupplied medication was relatively small, further analysis of data according to asthma medication supply status, under or over supply, made no difference to any of the observed outcomes.

## Discussion

### Summary of Main Findings

The findings from this study confirm and extend the results of previous studies which have reported poor adherence with asthma controller medications amongst children [7]–[8], [14]–[18]. However the relationship between low level asthma medication adherence and disease control appears complex with evidence to suggest that adequate MPR is associated with greater use of rescue medication.

### Strengths and Limitations of the Study

This study examined adherence levels in a large paediatric population in a “real world” primary care setting. The assessment of adherence with asthma medication is important and permits identification of patients requiring further intervention, and the evaluation of clinical outcomes associated with poor adherence. In addition, using this methodology adherence in a large population can be assessed without influencing patient behaviour and avoiding reporting and/or interviewer bias. Nevertheless, this study has several limitations: prescribing databases cannot confirm whether the medications were actually used. However, previous studies have reported that adherence rates, measured using healthcare databases, demonstrate high concordance with rates assessed by objective and accurate methods such as weighing inhalers, pill counting and/or electronic monitoring [19]–[20]. In the present study prescribed medications were used as a proxy for asthma diagnosis and severity assuming that medications were collected by patients and used as prescribed. The use of prescribing data may overestimate adherence in cases where the treatment has been intended by the prescriber to be used intermittently or seasonally. However, in this study, only children on the BTS step 2 or higher asthma regimes were included in the study population, therefore intermittent or seasonal use is unlikely to be a significant issue.

### Comparison with Existing Literature

As in previous reports, this study identified that the proportion of children with adequate MPR for controller medications was within the 11–28% reported by others [14]–[17] and that factors associated with poor adherence include low socioeconomic status, female gender and age greater than 12 years [19], [21]. Similarly, in this study the oral route was associated with significantly better adherence when compared to inhalational therapies [18], [22]–[24].

It has been previously reported that adherence rates tend to be lowest in patients prescribed multiple medications, and simplification of the treatment regimen by the use of combination inhalers should improve adherence [17]. However in this study combination therapy (LABA/ICS) was not associated with improved adherence and more children with adequate MPR were prescribed separate LABA+ICS than combined LABA/ICS. A similar finding was observed by Latry et al (2008), who reported that adults with asthma adhered less well to LABA and ICS treatment when it was delivered by a single inhaler than when it was delivered concurrently via two separate devices [25]. This is a surprising observation that seems to contradict the findings of previous observational database studies [17], [23], [26]–[28]. One possible explanation for this finding could be confounding by asthma severity as children with more severe asthma may be treated more aggressively and prescribed more LABA+ICS resulting in higher drug supply and hence higher MPR. A further possible explanation could be that patients on concurrent LABA+ICS may overuse the LABA component of the prescription, and then trigger a repeat prescription which is issued for both LABA and ICS resulting in an apparently greater MPR in this group. Finally, the apparent lower adherence associated with combination inhalers might be attributed to what has been termed “depletion of susceptibles”; that is patients identified by their GP as poorly adherent with separate ICS + LABA therapy may be prescribed a combined inhaler to improve their adherence, resulting in an apparently greater rate of adherence in the remaining subjects continuing to use ICS + LABA [25].

We expected to find a positive association between inadequate adherence and poor asthma control reflected by increased prescribing of rescue medication (OCS and SABA). However, in this study, children with adequate MPR were more likely to be prescribed OCS and/or six or more SABA canisters in the index year. Similar observations have been reported by others [13], [21], [24], [29]–[30]. The reasons for this paradoxical finding are not clear, but may indicate prescribing of an inadequate dose, or poor inhalational technique leading to poor asthma control despite optimum therapy [29], [31]. Other reasons may include lack of awareness of over prescribing of SABA and/or OCS in children with adequate control due to multiple prescribers, automated and telephone requests for repeat prescriptions [32] or that children with poorly controlled asthma may have required more aggressive treatment resulting in increased prescribing of asthma controllers and hence higher MPR [33].

### Implications for Future Research or Clinical Practice

The use of routinely acquired computerised prescribing data permits a “real world” assessment of adherence, predictive factors and possible outcomes. This study suggests that poor adherence to asthma controller medication is common in children. Furthermore poor asthma control, measured by requirement for rescue medication, was evident even in children with an adequate MPR.

The association between level of asthma control and adherence to controller medications does not appear to be a straightforward issue as patients may reduce their prescribed controller medication use without negative consequences [30] while others may continue to have poor outcomes despite optimum treatment [34]. The “minimum accepted” level of adherence with asthma medications to achieve control will remain a question.

Researchers should be aware of the challenges that can compromise the validity of findings from such studies and of various methodological approaches to address these possible shortcomings. However, despite limitations, prescribing data constitute an available, low cost method to assess adherence in large populations and thereby to identify patients with low adherence who may need further intervention to better manage their disease.
